# Ultrafast Charge Transfer Cascade in a Mixed-Dimensionality Nanoscale Trilayer

**DOI:** 10.1021/acsnano.3c12179

**Published:** 2024-03-11

**Authors:** Alexis R. Myers, Zhaodong Li, Melissa K. Gish, Justin D. Earley, Justin C. Johnson, M. Alejandra Hermosilla-Palacios, Jeffrey L. Blackburn

**Affiliations:** † National Renewable Energy Laboratory, Golden, Colorado 80401, United States; ‡ Department of Chemistry, University of Colorado−Boulder, Boulder, Colorado 80309, United States; § The Institute of Technological Sciences, Wuhan University, Wuhan, Hubei 430072, China

**Keywords:** transition metal dichalcogenides, charge transfer, heterojunctions, carbon nanotubes, excitons

## Abstract

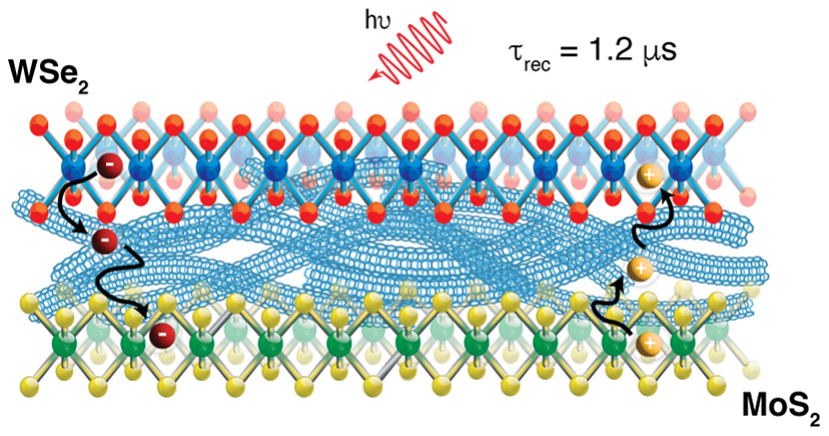

Innovation in optoelectronic semiconductor devices is driven by a fundamental understanding of how to move charges and/or excitons (electron–hole pairs) in specified directions for doing useful work, e.g., for making fuels or electricity. The diverse and tunable electronic and optical properties of two-dimensional (2D) transition metal dichalcogenides (TMDCs) and one-dimensional (1D) semiconducting single-walled carbon nanotubes (s-SWCNTs) make them good quantum confined model systems for fundamental studies of charge and exciton transfer across heterointerfaces. Here we demonstrate a mixed-dimensionality 2D/1D/2D MoS_2_/SWCNT/WSe_2_ heterotrilayer that enables ultrafast photoinduced exciton dissociation, followed by charge diffusion and slow recombination. Importantly, the heterotrilayer serves to double charge carrier yield relative to a MoS_2_/SWCNT heterobilayer and also demonstrates the ability of the separated charges to overcome interlayer exciton binding energies to diffuse from one TMDC/SWCNT interface to the other 2D/1D interface, resulting in Coulombically unbound charges. Interestingly, the heterotrilayer also appears to enable efficient hole transfer from SWCNTs to WSe_2_, which is not observed in the identically prepared WSe_2_/SWCNT heterobilayer, suggesting that increasing the complexity of nanoscale trilayers may modify dynamic pathways. Our work suggests ”mixed-dimensionality” TMDC/SWCNT based heterotrilayers as both interesting model systems for mechanistic studies of carrier dynamics at nanoscale heterointerfaces and for potential applications in advanced optoelectronic systems.

Optoelectronic semiconductor devices rely on the balance between photoexcited charge generation, charge recombination, and charge extraction.^1−3^ Quantum-confined low-dimensional materials such as two-dimensional (2D) transition metal dichalcogenides (TMDCs) and one-dimensional (1D) single-walled carbon nanotubes (SWCNTs) have enhanced electron–hole Coulomb interactions, weak dielectric screening, and large exciton binding energies.^3−12^ Large exciton binding energies can correlate with short exciton lifetimes and increased difficulty in separating electrons and holes to generate an electrical current for photovoltaics, photodetectors, and sensors or chemical bonds in solar fuel schemes.^2,3,8,11−13^ The diverse and tunable electronic and optical properties of TMDCs and semiconducting SWCNTs (s-SWCNTs) make them good quantum-confined model systems for fundamental studies on charge and exciton transfer across heterointerfaces, with relevance for applications in photovoltaics, quantum information processing, and solar fuels.^3,5,7,10,14−16^ Recent studies of TMDC heterobilayers have shown charge recombination lifetimes of ca. 30–110 ps, and TMDC/organic heterojunctions have achieved a charge separated state lifetime of ca. 5 ns in a MoS_2_/pentacene bilayer, demonstrating that charge separation in 2D heterostructures is an efficient strategy to overcome large exciton binding energies.^3,8,17^

In analogy to the multistep charge transfer cascade found in the photosynthesis reaction center, more complex low-dimensional heterostructures provide opportunities for directing charge flow and lengthening charge separation lifetimes.^13,18^ For example, TMDC hetero-*trilayer* interfaces have even longer lifetimes, up to ca. 1 ns, than their heterobilayer counterparts. As these heterostructures become more complex, it can be difficult to predict the range of motion and mechanism(s) for generating long-lived charges across the relevant interfaces.^1,3,5,8,14,16^ For example, in an MoS_2_/WS_2_/MoSe_2_ Type II heterotrilayer, Ceballos et al. predicted a cascading charge transfer effect, where photoexcited electrons in MoSe_2_ would transfer to MoS_2_ via WS_2_.^19^ However, electrons excited in MoSe_2_ transferred directly to MoS_2_ without populating the WS_2_. Furthermore, the seemingly ubiquitous existence of Coulomb-bound “interlayer” excitons in TMDC heterostructures, while useful for certain applications, can limit the ultimate lifetime of separated charges and impede the generation and extraction of free uncorrelated electrons and holes.

Sulas-Kern et al. recently demonstrated that a “mixed-dimensionality” heterobilayer combining TMDCs with s-SWCNTs resulted in charge-separated lifetimes on the *microsecond* time scale, surpassing TMDC/TMDC and TMDC/organic charge lifetimes.^3,5,19−21^ Despite the success of this heterostructure in producing exceptionally long-lived charge carriers, fundamental questions still exist such as (1) if and/or how the electron–hole pair at the donor–acceptor interface can escape from the mutual “interlayer” Coulombic attraction and (2) the roles both in and out of plane carrier delocalization and/or diffusion have on stabilizing long-lived charges.^3,19,22,23^ Here we use transient absorption (TA) spectroscopy to track exciton dissociation and charge diffusion in a mixed-dimensionality 2D/1D/2D MoS_2_/SWCNT/WSe_2_ heterotrilayer. Upon photoexcitation of the trilayer, we observe ultrafast electron transfer to MoS_2_ and hole transfer to WSe_2_, with microsecond charge recombination lifetimes. Interestingly, the trilayer architecture appears to facilitate ultrafast hole transfer to WSe_2_ (<200 fs), whereas this charge transfer reaction is not as efficient in identically prepared WSe_2_/SWCNT heterobilayers. By tracking multiple well-resolved spectral signatures of charge carriers in each semiconductor layer, we can experimentally monitor and simulate charge diffusion away from the site of charge generation, confirming the separation of electron–hole pairs at the TMDC/SWCNT interface and subsequent diffusion to the other 2D/1D interface. This behavior contrasts with the dominance of Coulomb-bound “interlayer” excitons in TMDC/TMDC heterobilayers or trilayers. Our results highlight the distinct photophysics and potential technological advantages of “mixed-dimensionality” heterostructures.

## Results and Discussion

### MoS_2_ and WSe_2_ Bilayers

**Figure 1:**
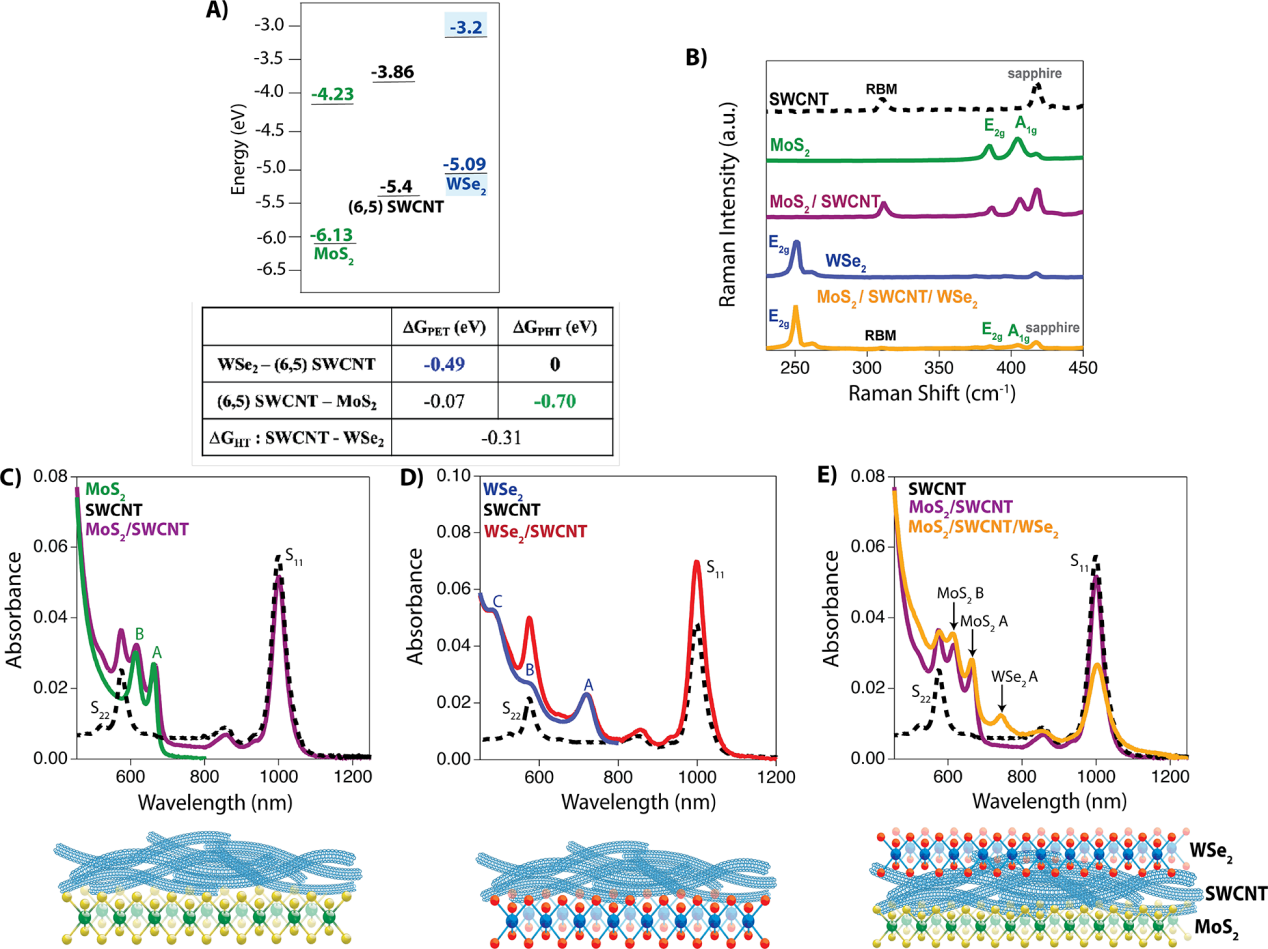
(A) Predicted energy level diagram of MoS_2_/SWCNT and WSe_2_/SWCNT bilayers (top); Calculated thermodynamic driving forces for electron and hole transfer at the MoS_2_/SWCNT and WSe_2_/SWCNT interfaces (bottom). (B) Raman spectra of (6,5) SWCNT film, MoS_2_ monolayer, MoS_2_/SWCNT bilayer, WSe_2_ monolayer, and the MoS_2_/SWCNT/WSe_2_ trilayer. (C) Absorbance spectra for MoS_2_ monolayer, (6,5) SWCNT film, and the MoS_2_/SWCNT heterojunction (top); Schematic of MoS_2_/SWCNT bilayer (bottom). (D) Absorbance spectra of WSe_2_ monolayer, (6,5) SWCNT film, and the WSe_2_/SWCNT bilayer (top); Schematic of the WSe_2_/SWCNT bilayer. (E) Absorbance spectra of (6,5) SWCNT film, MoS_2_/SWCNT bilayer, and MoS_2_/SWCNT/WSe_2_ trilayer (top); Schematic of the MoS_2_/SWCNT/WSe_2_ trilayer (bottom).

To understand the exciton and charge dynamics in our MoS_2_/SWCNT/WSe_2_ heterotrilayer, we first studied individual bilayers made from combining s-SWCNTs with each TMDC monolayer. Monolayer TMDCs are used throughout this paper and characterized via Raman spectroscopy; see Figure 1c–e. Both MoS_2_/SWCNT and WSe_2_/SWCNT bilayers were obtained by spray coating a thin film of (6,5) SWCNTs onto the surface of the respective neat TMDC monolayer.^1,16,24^ The (6,5) SWCNT film thickness was estimated to be ca. 7 nm.^16,24^ Steady state absorption and Raman spectroscopy were used to confirm the successful assembly of both bilayers (Figure 1c,d).^5,25−32^

The thermodynamic driving force for free carrier generation depends on the free energy difference between initially photoexcited excitons and the final separated charge carriers (electron and hole). In a type II heterojunction, the “donor” has an electron affinity and ionization potential closer to vacuum than the “acceptor”.^3^Figure 1a depicts the expected Type II heterojunction energy level offset for the MoS_2_/SWCNT and WSe_2_/SWCNT bilayers, where the conduction band (CB) and valence band (VB) energies of MoS_2_ and WSe_2_ are taken from theoretical and experimental literature reports (*vide infra* and SI, Table S3). We estimate the exciton dissociation driving force for each Type II bilayer using the following equation:
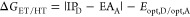
1where Δ*G*_ET/HT_ is the change in free energy following electron transfer (ET) or hole transfer (HT), IP_D_ and EA_A_ are the ionization potential (IP) of the donor and electron affinity (EA) of the acceptor, respectively, and *E*_opt,D_ and *E*_opt,A_ refer to the optical band gap of the donor or acceptor, respectively.^3,16^ Using optical band gap energies from absorption spectra (Figure 1c–e) and EA/IP values shown in Figure 1a, we estimate that exciton dissociation in MoS_2_ via hole transfer to (6,5) SWCNTs is thermodynamically favorable by ca. 700 meV and electron transfer from the SWCNTs to MoS_2_ is favorable by ca. 70 meV.^3,15,16^ Uncertainty in Δ*G*_ET_ and Δ*G*_HT_ for WSe_2_ is higher due to slight variations in the wavelength for the WSe_2_ A exciton peak across different samples and a higher variability (and smaller number) of reported experimental and computed CB/VB values of WSe_2_ compared to the more widely studied MoS_2_.^7^ To account for these variations, we used experimental data captured from steady state absorption to calculate *E*_opt,WSe_2__, resulting in a Δ*G*_ET_ of −490 meV and a Δ*G*_HT_ of 0 meV. Theoretically, exciton dissociation at the interface can occur for both bilayers in both directions, but the hole transfer from (6,5) s-SWCNTs to WSe_2_ is the least energetically favorable pathway.

Transient absorption (TA) spectroscopy is used to elucidate the charge transfer dynamics at the MoS_2_/SWCNT and WSe_2_/SWCNT interfaces. The narrow and energetically distinct absorbance peaks of MoS_2_, WSe_2_ and (6,5) SWCNTs, highlighted in Figure 1e, allow for selective photoexcitation and probing of each material, making these ideal model systems for optical studies of excited-state dynamics at the TMDC heterointerface. Photoexcitation of the SWCNT S_11_ absorption at 1000 nm only allows for charge transfer from SWCNT to either/both TMDC, as the 1000 nm photons are too low in energy for direct excitation of MoS_2_ or WSe_2_ and energy transfer would be significantly uphill.

**Figure 2:**
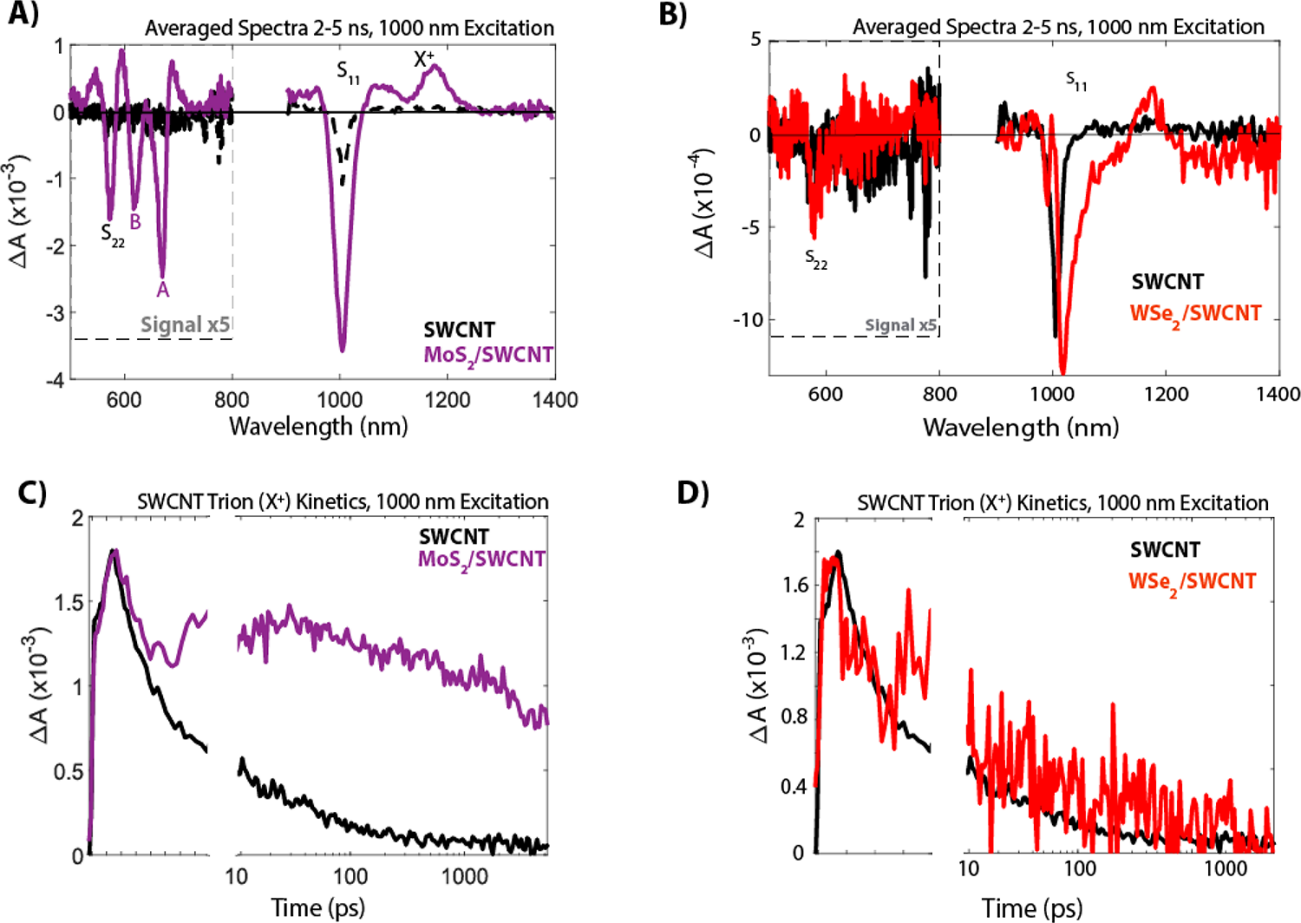
Transient absorption spectra averaged over 2–5 ns following 1000 nm excitation of (A) SWCNT (black) and MoS_2_/SWCNT bilayer (purple) and (B) SWCNT (black) and WSe_2_/SWCNT bilayer (red). Kinetic traces corresponding to the SWCNT trion (X^+^ or X^–^, depending on the transferred charge) induced absorption with 1000 nm excitation: (C) SWCNT (black) and MoS_2_/SWCNT bilayer (purple) and (D) SWCNT (black) and WSe_2_/SWCNT bilayer (red).

Figure 2 shows long-lived TA spectra of both bilayers following 1000 nm excitation of the s-SWCNT S_11_ excitonic transition. In the MoS_2_/SWCNT bilayer, we see charge separation that persists well beyond the 5 ns window of the TA experiment, consistent with previous MoS_2_/SWCNT studies, the Type II energetic alignment and the thermodynamic spontaneity for photoinduced electron transfer expected from Figure 1a.^3,5,25^ In the near-infrared (NIR) range of photoexcited neat MoS_2_ monolayers there is no TA signal, since all MoS_2_ excitonic features occur in the visible range. However, clear signatures of an SWCNT charge population in the MoS_2_/SWCNT bilayer indicate photoinduced electron transfer from SWCNTs to MoS_2_ (Figure 2a). The well-documented signature is the SWCNT trion (X^+^) induced absorption (IA) at 1175 nm and the accompanying 1000 nm ground-state bleach (GSB) of the S_11_ transition.^5,16^ These NIR features are complemented by long-lived features in the visible range, which we can attribute to the MoS_2_ A and B excitons (bleaches at 660 and 610 nm, respectively) and to the SWCNT S_22_ transition (575 nm bleach). Figure 2c demonstrates long-lived carrier separation in the MoS_2_ heterobilayer compared to the neat SWCNTs via charge-related kinetics at the 1175 nm SWCNT trion-induced absorption. We note the presence of a second pulse in the TA kinetics, around 10 ps, due to reflection off of the air-free holder sapphire windows. This second pulse is present in all our samples and it should not be mistaken for a delayed rise of a given population. The effect of the second pulse is explained and addressed in the Supporting Information.

In contrast to the MoS_2_/SWCNT bilayer, when exciting the WSe_2_/SWCNT bilayer at 1000 nm, the WSe_2_ GSB at 750 nm is absent in the TA spectra (Figure 2b), indicating that there is negligible (or low-yield) exciton dissociation via hole transfer from the SWCNTs to WSe_2_. However, a small trion feature is observed in the NIR region, from which we calculated a hole transfer yield of 2% after 1000 nm excitation. This low hole transfer yield could explain why we do not observe the WSe_2_ GSB at 750 nm, since the GSB could be embedded in the noise. The kinetics at 1175 nm change very little relative to that of the neat SWCNT, also suggesting negligible charge generation in this bilayer. This inefficient hole transfer event may result from the low thermodynamic driving force of ca. 0 meV (Figure 1a), even though the calculated band alignment is Type II. We confirmed the absence of the WSe_2_ GSB at 750 nm after 1000 nm excitation in this bilayer through TA experiments on two other separately prepared bilayers that incorporated CVD-grown WSe_2_ monolayers that were either purchased or grown in-house at NREL (Figure S1).

Interestingly, we also observe a relatively low charge generation yield in the WSe_2_/SWCNT bilayer after photoexcitation of the WSe_2_ layer at 750 nm, approximately 10% (Figure S1). We speculate on two potential mechanisms for the somewhat low efficiency of this electron transfer event. It is possible that the large predicted thermodynamic driving force of ca. 490 meV (Figure 1a) places this electron transfer event in the Marcus inverted regime,^9^ although hole transfer from MoS_2_ to (6,5) s-SWCNTs is substantially more efficient (ca. 39% with Δ*G*_HT_ = −700 meV). Fang and co-workers have also proposed the formation of interlayer excitons in WSe_2_/SWCNT heterojunctions, based on PL features observed at room temperature.^33^ It is unclear how these interlayer excitons would manifest in our TA measurements, so this subject will be considered in future studies.

### MoS_2_/SWCNT/WSe_2_ Trilayer

**Figure 3:**
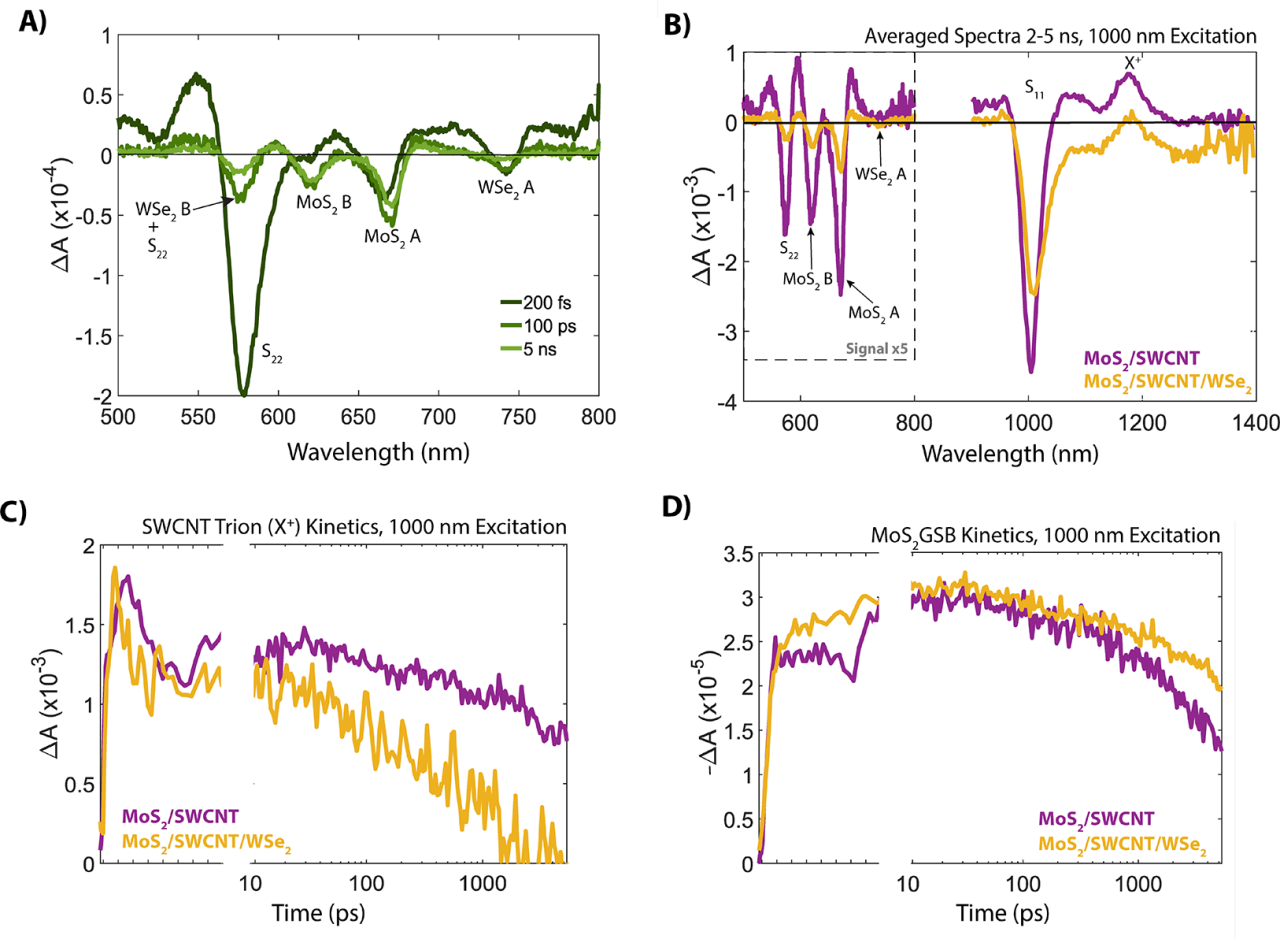
(A) Transient absorption spectra, at varying pump-probe time delays, for the MoS_2_/SWCNT/WSe_2_ trilayer, following 1000 nm excitation. (B) Transient absorption spectra averaged over 2–5 ns for the MoS_2_/SWCNT bilayer (purple), and MoS_2_/SWCNT/WSe_2_ trilayer (orange). (C) and (D) Kinetic traces at (C) 1175 nm, corresponding to the SWCNT trion (X^+^) induced absorption, and (D) 660 nm, corresponding to the ground state bleach of MoS_2_, following 1000 nm excitation.

We prepared the MoS_2_/SWCNT/WSe_2_ trilayer by transferring a CVD-grown WSe_2_ monolayer onto the existing MoS_2_/SWCNT bilayer.^34−36^Figure 1b,e shows Raman and steady state absorption spectra comparing MoS_2_, the MoS_2_/SWCNT bilayer, and the MoS_2_/SWCNT/WSe_2_ trilayer. The peak at 250 cm^–1^ in the trilayer Raman spectrum is the WSe_2_ out-of-plane (*E*_2*g*_) Raman active mode and in the trilayer absorption spectra, the WSe_2_ A exciton peak appears at 750 nm, indicating the successful creation of the heterotrilayer.^26,28^ TA spectroscopy of the trilayer shows ultrafast exciton dissociation at both TMDC/SWCNT interfaces and a resulting long-lived charge separation. Figure 3a highlights the formation of separated carriers within femtoseconds (<200 fs) following 1000 nm excitation, evidenced by the observation of both WSe_2_ and MoS_2_ bleach features, followed by charge recombination that persists beyond the nanosecond time scale.

Figure 3a,b demonstrates that not only does photoinduced hole transfer from MoS_2_ to SWCNTs occur in the trilayer (similar to what is observed for the MoS_2_ bilayer), but the electron transfer event from WSe_2_ to SWCNTs, which is inefficient for the WSe_2_ bilayer, is observed in the trilayer. This unexpected result suggests that some ground- or excited-state properties of the trilayer change the spontaneity/efficiency of the photoinduced hole transfer event from (6,5) SWCNTs to WSe_2_, relative to the corresponding WSe_2_/SWCNT bilayer. We speculated that releasing WSe_2_ from its original sapphire substrate may modify the VB energy in such a way that HT is now spontaneous in the trilayer but not in the bilayer. However, photoexcitation of a “reverse bilayer” (SWCNT/WSe_2_), in which the SWCNTs are sprayed onto WSe_2_ that remains on its original substrate, refutes this idea as hole transfer from SWCNT to WSe_2_ is not present in the transient data (Figure S2).

The photoinduced charge transfer quantum yield (ϕ _CT_) is defined as
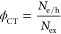
2where *N*_e/h_ is the number of separated charges (electrons or holes) per photogenerated exciton (*N*_ex_).^1,5,25^ Using the trion-induced absorption intensity as in prior studies, we estimate the charge yield in the trilayer to be 41%, which is a summation of electron and hole transfer yields at the MoS_2_ and WSe_2_ interfaces, respectively.^37^ The addition of the hole-accepting WSe_2_ layer to MoS_2_/SWCNT roughly doubles the total charge transfer yield when compared to the 23% electron transfer yield in the MoS_2_/SWCNT bilayer.^5^ This result positions the trilayer as an effective method for significantly increasing the charge yield.

Comparing the kinetics of specific spectral features between the MoS_2_/SWCNT bilayer and the MoS_2_/SWCNT/WSe_2_ trilayer can provide a window into the diffusive charge dynamics that occur following photoinduced exciton dissociation in the trilayer. In Figure 3c,d, pumping at 1000 nm excites only the SWCNTs, generating both the MoS_2_(−)/SWCNT(+) and WSe_2_(+)/SWCNT(−) interfacial species. Figure 3c compares the kinetics of the SWCNT trion induced absorption in the trilayer and the MoS_2_/SWCNT bilayer. In the trilayer, the trion transition gets its oscillator strength from both holes (i.e., X^+^) at the MoS_2_(−)/SWCNT(+) interface and electrons (i.e., X^–^) at the WSe_2_(+)/SWCNT(−) interface. In the MoS_2_/SWCNT bilayer, the X^+^ signal is long-lived and decays outside of the nanosecond window, while in the trilayer the trion signal (X^+^ + X^–^) is substantially reduced by 5 ns. This depletion of SWCNT charges could arise from two diffusive processes: (1) diffusion-limited recombination of electrons from the WSe_2_(+)/SWCNT(−) interface with holes from the MoS_2_(−)/SWCNT(+) interface within the SWCNTs; (2) diffusion of electrons away from the WSe_2_(+)/SWCNT(−) interface to the MoS_2_(−)/SWCNT(+) interface where they are injected into MoS_2_ (and/or the reverse process for holes at the MoS_2_(−)/SWCNT(+) interface). Both of these processes require SWCNT-based carriers at the SWCNT/TMDC interface to escape the Coulomb potential of the oppositely charged TMDC-based carrier.

To determine if process (2) from above is active, we track the MoS_2_ GSB kinetics at 660 nm (Figure 3d). 1000 nm is used unless otherwise stated. Encouragingly, the MoS_2_ GSB does not decay as rapidly in the trilayer, relative to the MoS_2_/SWCNT bilayer, suggesting that this diffusion-based process may be occurring. Thus, the concomitant depletion of the trion IA and increase of the MoS_2_ GSB in the trilayer provide evidence for charges escaping the WSe_2_–SWCNT interface and diffusing to the MoS_2_–SWCNT interface where they inject into MoS_2_. When tracking the MoS_2_ GSB, selective excitation of WSe_2_ in the trilayer results in a slow rise of MoS_2_ charges, also supporting this diffusion based process (see Figure S4).

**Figure 4:**
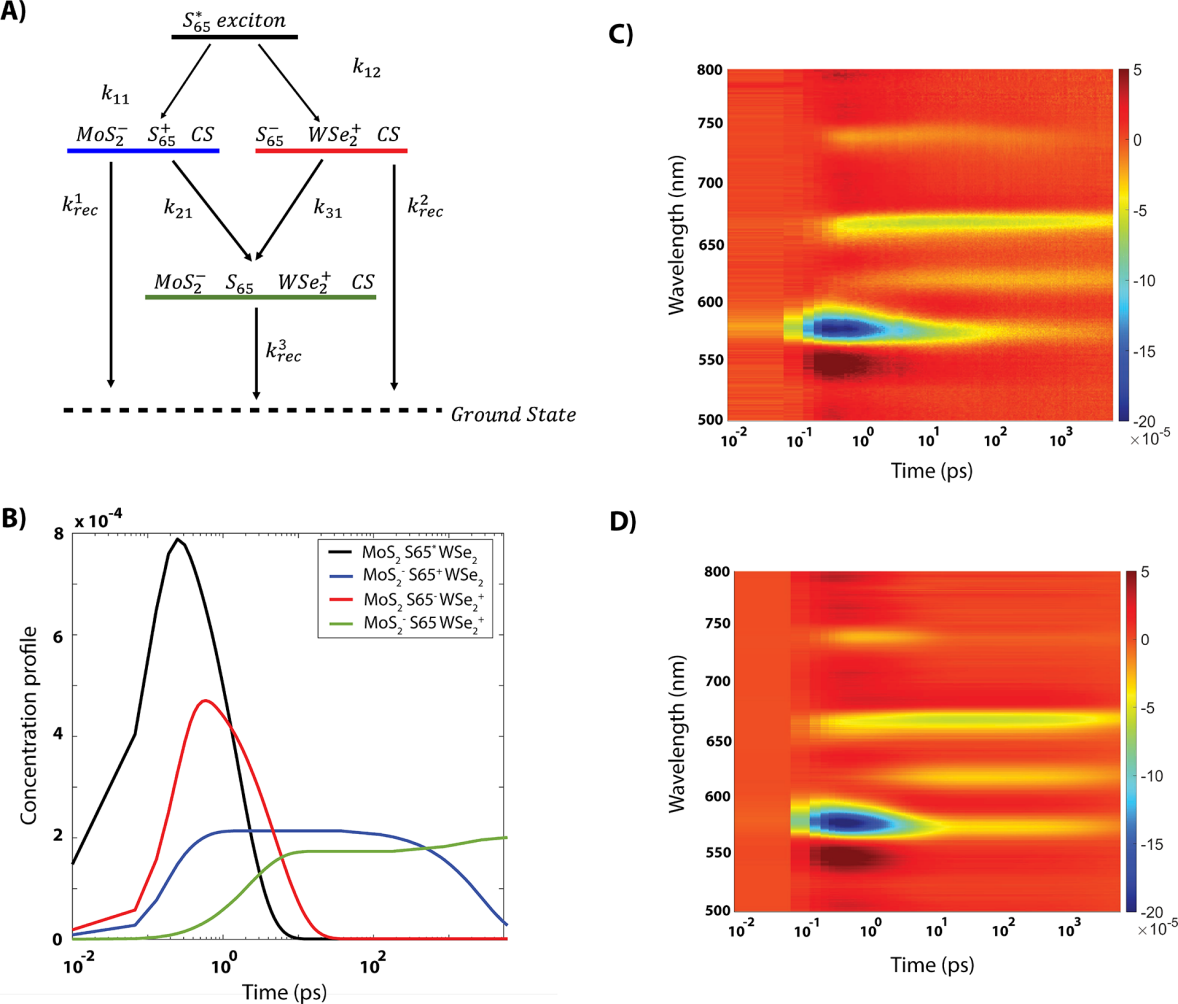
(A) Proposed kinetic scheme, following SWCNT excitatoin at 1000 nm; (B) Concentration profiles for each species generated in the trilayer, with similar color-coding to panel (A); (C) Experimental TA surface plot for the visible region of the trilayer excited at 1000 nm; (D) Simulated TA surface plot from the concentration equations and associated spectra. The color bar to the right specifies intensities of the different signals. Specifically, the GSB for S_22_ at 575 nm, MoS_2_ A exciton at 660 nm and WSe_2_ A excitonat 740 nm can be identified. Color bar to the right specifies intensities of the different signals.

**Figure 5:**
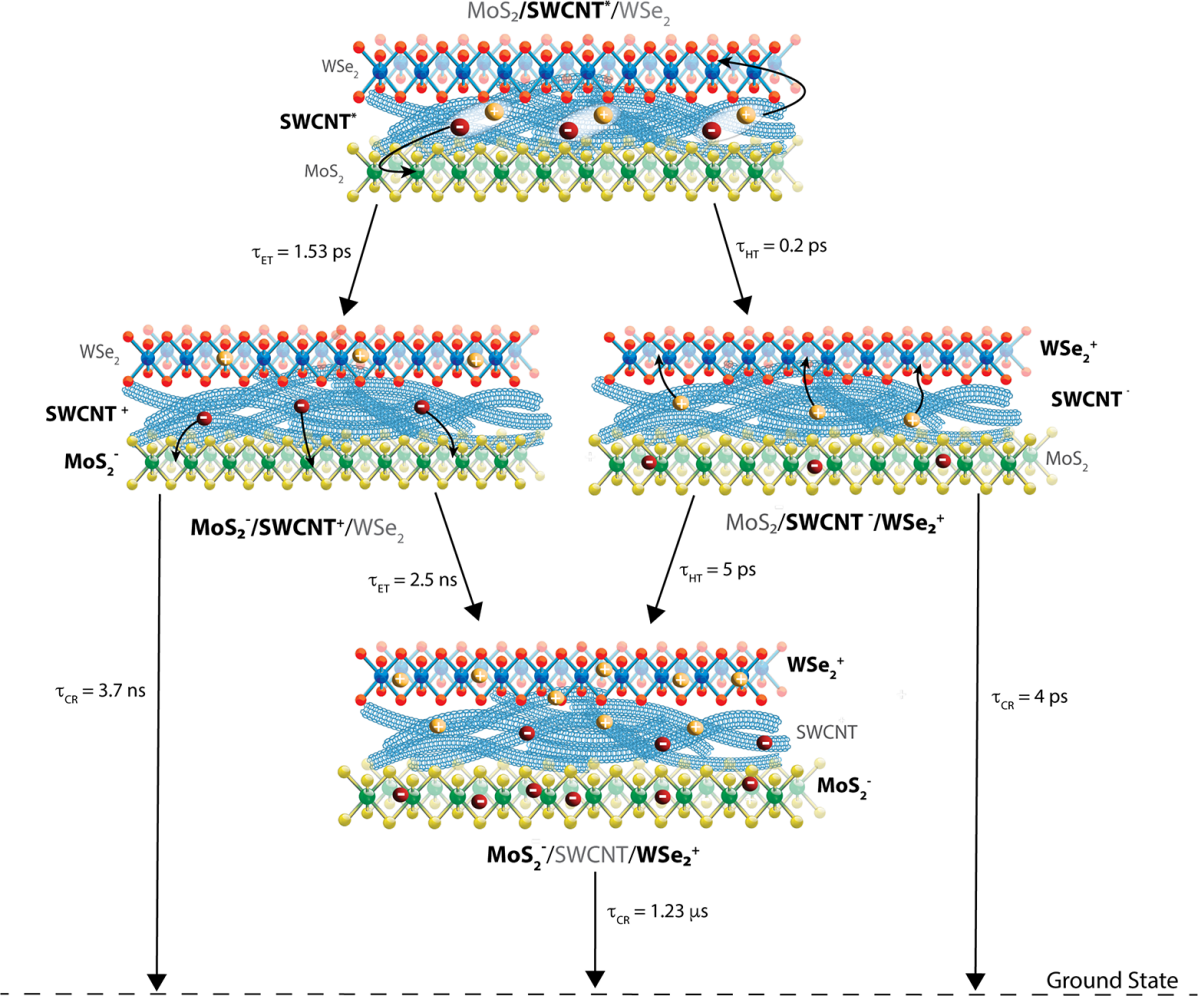
Kinetic scheme highlighting the different time constants for hole transfer (τ_HT_) to WSe_2_, electron transfer (τ_ET_) to MoS_2_ and the charge recombination lifetimes (τ_CR_) following selective excitation of SWCNT at 1000 nm.

To confirm that charge carrier diffusion plays a role after exciton dissociation and to elaborate on the overall kinetic scheme of this complex system, we turn to a detailed model of the exciton and charge dynamics. Experimental time-dependent spectral contributions of excitons and charges are separated using singular value decomposition (SVD).^38,39^ Based on initial TA information, four independent species are used in SVD analysis to extract the decay of charge-related spectral signatures (Figure S5): (1) the initial SWCNT excited state created by excitation at 1000 nm (S65*); (2) electron transfer from SWCNT to MoS_2_ (S65^+^ MoS_2_^–^); (3) hole transfer from SWCNT to WSe_2_ (S65^–^ WSe_2_^+^); and (4) charge (hole/electron) on s-SWCNT diffusing away to corresponding TMDC (WSe_2_^+^/MoS_2_^–^), with opposite charges on TMDCs remaining on the respective layer. Figure 4a displays the kinetic scheme that ultimately simulates the 2D TA data most effectively, with the rate equations associated with this scheme provided in Supporting Information secton 1.3. The kinetic scheme and derived equations from the simulation can accurately reproduce the 2D experimental data (Figure 4c,d). Time scales for electron transfer (*k*_11_), hole transfer (*k*_22_), diffusion (*k*_21_ and *k*_31_), and each charge recombination (*k*_rec_) pathway are obtained from the simulated concentration profiles in Figure 4b and are outlined in the visual schematic in Figure 5 (see SI for additional information).

Despite a greater predicted thermodynamic driving force for electron transfer (see Figure 1a, Δ*G*_ET_ = −70 meV), we observe faster hole transfer from the SWCNTs to WSe_2_ (τ_HT_ = <0.2 ps) compared to electron transfer from the SWCNTs to MoS_2_ following selective excitation at 1000 nm (τ_ET_ = 1.53 ps). Subsequently, diffusion of holes away from the SWCNT/WSe_2_ interface also occurs at a faster rate (τ_HT_ = 5 ps) than diffusion of electrons away from the SWCNT/MoS_2_ interface (τ_ET_ = 2.5 ns). Charge recombination of both holes and electrons successfully transferred from SWCNTs to either WSe_2_ or MoS_2_ occurs with a time constant of 1.23 μs. The trilayer nearly doubles the charge recombination time, relative to the MoS_2_/SWCNT bilayer (τ_rec_ = 0.73 μs, see also Figure S4).^5^ Thus, improved spatial separation in the TMDC/SWCNT/TMDC trilayer leads to long charge recombination lifetimes.^5,8,17^

Our study was motivated by the natural charge transfer cascades driving long-lived charge separation in photosynthesis, as well as a lack of mechanistic understanding of carrier diffusion at TMDC heterointerfaces. The TA analysis on this mixed-dimensionality heterostructure reveals several intriguing features that warrant some discussion. First, the MoS_2_/SWCNT/WSe_2_ trilayer allows us to access the HT pathway that is ineffective in the respective bilayer. As prepared, MoS_2_ tends to be n-type, while WSe_2_ is typically p-type as-grown. We speculate that having the two oppositely charged layers on either side of the s-SWCNT film may lead to the formation of an electric field or dipole that shifts the band alignment, making HT more favorable in the trilayer than in the bilayer.^2,17^ Second, we demonstrate that charges within the s-SWCNT layer can escape the Coulomb attraction of the opposite charge carrier at the SWCNT/TMDC interface. In contrast, charge transfer in similar TMDC/TMDC heterojunctions is typically characterized by spatially confined and tightly bound interfacial excitons with limited mobility away from the heterointerface. The faster time scale for this diffusive process at the WSe_2_/SWCNT interface, relative to the MoS_2_/SWCNT interface, may suggest a stronger interlayer Coulomb binding energy at the MoS_2_/SWCNT interface. We plan to probe these interesting observations in more detail, both experimentally and theoretically, in future studies.

## Conclusion

In conclusion, we have successfully prepared a MoS_2_/SWCNT/WSe_2_ heterotrilayer that can separate charges via a charge transfer cascade. By using transient absorption spectroscopy, we have shown that this type of “mixed-dimensionality” Type-II trilayer can yield charge separated lifetimes on the microsecond time scale while significantly increasing charge yield relative to similarly prepared Type-II bilayers. The microsecond charge recombination lifetime demonstrates the significance of SWCNTs as the charge carrier medium and the role that out-of-plane carrier delocalization has on stabilizing long-lived charges. Consistently, the well-resolved SWCNT spectral signatures allowed us to track charge diffusion away from the site of charge generation and demonstrate Coulombically unbound charges moving from one TMDC/SWCNT interface to the other. The observation of a charge transfer pathway in the trilayer that is ineffective in the respective WSe_2_/SWCNT bilayer suggests that strategic layering in nanoscale heterojunctions provides additional routes for charge movement in specified directions. Ultimately, our results suggest that well-defined charge transfer cascades can result in longer charge separated lifetimes and higher yields of e^–^/h^+^ pairs that can escape their mutual Coulombic attraction, positioning these nanoscale model systems as interesting for both fundamental studies and optoelectronic devices.

## Methods

### TMDCs

Full coverage CVD grown monolayer MoS_2_ and WSe_2_ on c-cut sapphire were purchased from 2D semiconductors. The NREL grown monolayer WSe_2_ was prepared via chemical vapor deposition (CVD) in a three-temperature zone furnace. An alumina boat positioned 1 in. from zone 1 (upstream, outside of the furnace) contained 500 mg of selenium, and zone 3 contained a Si/SiO_2_ wafer with a predeposited thin film of 10 mM ammonium metatungstate (AMT) and 10 mM NaOH. 100 sccm aliquot of Ar/2% H_2_ gas was supplied to the growth chamber to carry the selenium under a growth pressure of 760 Torr. Zone 1, 2, 3 had a temperature ramping rate of 35 °C/min and were heated to 530, 950, and 950 °C, respectively, and maintained for 4 min.

### Absorption

Ground state absorption spectra of both neat and bilayer films were measured in air on a Cary 5000 spectrophotometer with baseline correction. The (6,5) SWCNT film thickness was estimated to be ca. 7 nm from the S_11_ peak optical density at 1000 nm.

### Raman and Photoluminescence Spectroscopy

Measurements were taken in an air-free cell using an inVia Renishaw confocal Raman microscope with a 532 and 633 nm laser.^40^ Raman scattering was detected using a 1800 lines/mm grating, and photoluminescence (PL) was recorded using a 600 lines/mm grating. A GaInP/AlGaInP sample was used as a reference to account for the day to day changes in laser power. All single Raman and PL spectra, e.g., in Figure 1, are averages of 50–100 spectra taken over a specific area of each sample and then normalized to the GaInP/AlGaInP reference.

### Transient Absorption Spectroscopy

Samples for transient absorption measurements were sealed inside a nitrogen glovebox in a double window air-free sample holder to prevent exposure to the atmosphere and avoid sample degradation during TA measurements.

#### Ultrafast TA

Ultrafast TA was performed on a Coherent Libra Ti/Sapphire laser with a 1 kHz 800 nm output and 150 fs fwhm pulse width. The Coherent Libra output was sent through a beam splitter to generate the pump and probe. The pump was generated with a TOPAS-C optical parametric amplifier. Visible (450–800 nm) and NIR (800–1600 nm) probe pulses were generated by focusing the 800 nm fundamental into a thin or thick sapphire window, respectively. The TA was a pump–probe configuration measurement, contained in a HELIOS optical box from Ultrafast Systems controlled with a HELIOS software package. The probe beam is sent through a beam splitter to measure the reference spectrum to increase signal-to-noise ratio. The probe pulse, an 800 nm fundamental, is delayed with respect to the pump pulse using a mechanical delay stage. Data were chirp corrected and analyzed using a Surface Xplorer from Ultrafast Systems.

#### Nanosecond–Microsecond (100 ps to 400 μs) TA

Nanosecond–microsecond TA measurements were performed on a Coherent Libra Ti/Sapphire laser with a 1 kHz 800 nm output and 150 fs fwhm pulse width. The fundamental beam was used to generate pump pulses with a TOPAS-C optical parametric amplifier. Visible and NIR probe pulses are generated from an EOS (Ultrafast Systems) apparatus, produced in a diode-laser-pumped photonic crystal fiber, and electronically delayed relative to the pump pulse with a digital delay generator. Data acquisition was performed using the EOS software package from Ultrafast Systems.

### SWCNT Preparation

(6,5) Single-walled carbon nanotubes (SWCNTs) dispersions were prepared from SG65i SWCNTs (CoMoCAT, purchased from CHASM) and poly-[(9,9-dioctylfluorenyl-2,7-diyl)-*alt*-*co*-(6,60-[2,20-bipyridine])] (PFO-bpy) purchased from American Dye Source. Pure (6,5) SWCNTs were extracted from a solution of 0.5 mg/mL SWCNTs and 2 mg/mL of PFO-bpy in toluene via tip sonication (Cole-Palmer CPX 750, 0.5 in. tip) for 15 min at 40% amplitude in a flowing bath of cool water. Immediately after sonication, the dispersion was centrifuged at 13000 rpm for 5 min at 20 °C (Beckman Coulter L-100 XP ultracentrifuge, SW-32 Ti rotor). To remove excess polymer, the supernatant was separated from the pellet and centrifuged for 20 h at 24100 rpm and 20 °C. A compact pellet containing (6,5) SWNCTs and excess PFO-Bpy was redispersed in toluene in an ultrasonic bath sonicator for over an hour. The polymer removal process was then repeated for a second time.

### Spray Coating SWCNTs

MoS_2_/SWCNT and WSe_2_/SWCNT bilayers were prepared by spray coating the SWCNT ink onto the respective neat substrate. The substrates were placed on a heated metal stage (130 °C) to evaporate the toluene solvent. The ink was applied from an ultrasonic sprayer (SonoTek) directed at the substrate, operated at 0.8 W with a solution flow rate of 0.3 mL/min and a nitrogen flow rate of 7.0 standard liters per minute. Each bilayer film was soaked in their respective hot (78 °C) toluene baths for 10 min to remove additional polymer from the film.

### WSe_2_ Transfer

To transfer the WSe_2_ monolayer off of its sapphire growth substrate and onto the MoS_2_/SWCNT bilayer, the WSe_2_/sapphire substrate was coated with polystyrene (PS) dissolved in toluene (50 mg/mL) via spin coating at 2400 rpm for 45 s. Immediately after spin coating, all 4 sides of the film were scribed using a box cutter to create an opening for etchant to access the WSe_2_/sapphire interface. The substrate was placed in a hot (70 °C) 2 M NaOH bath for 10 min. The substrate was removed from the NaOH bath and the PS/WSe_2_ film detached in water by holding the substrate at the air/water interface. The PS/WSe_2_ film was transferred to the MoS_2_/SWCNT bilayer by lifting the substrate from underneath the film. The trilayer was then soaked in toluene for 10 min to remove the PS layer. This process was repeated for a second transfer. The film was placed in a spot different from the original transfer to maximize WSe_2_ surface coverage.

## Supporting Information Available

Additional transient absorption spectroscopy, kinetic analysis of trilayer, Δ*G* calculation values, and charge transfer yield calculations (PDF)

## Related Paper

Myers et al. Ultrafast Charge Transfer Cascade in a Mixed-Dimensionality Nanoscale Trilayer. *ChemRxiv*. **2023**. https://chemrxiv.org/engage/chemrxiv/article-details/656bf19dcf8b3c3cd7a95cdb (accessed February 15, 2024).
