# Supplementary material for: Ultrafast Charge Transfer Cascade in a Mixed-Dimensionality Nanoscale Trilayer
Source: ACS Nano. 2024 Mar 11;18(11):8190–8. doi: 10.1021/acsnano.3c12179 (PMC10958597; doi:10.1021/acsnano.3c12179)
Supplement: Supplementary file 1 — nn3c12179_si_001.pdf [file nn3c12179_si_001.pdf]

Supplementary Information for:
Ultrafast Charge Transfer Cascade in a Mixed-Dimensionality Nanoscale Trilayer

Alexis R. Myers,[†,‡] Zhaodong Li,[†,¶] Melissa K. Gish,[†] Justin D. Early,[†,‡] Justin C. Johnson,[†] M. Alejandra Hermosilla-Palacios[†]* and Jeffrey L. Blackburn[†]*

[†]National Renewable Energy Laboratory, Golden Colorado, 80401, USA
[‡]University of Colorado Boulder, Department of Chemistry, Boulder Colorado, 80309, USA
[¶]The Institute of Technological Sciences, Wuhan University, Wuhan, Hubei 430072, China

*Corresponding author email: alejandra.hermosillapalacios@nrel.gov, jeffrey.blackburn@nrel.gov

1 Transient Absorption Spectroscopy

1.1 Beam Size Measurements

The beam size for different pump excitation wavelengths was performed using a CCD camera beam profiler BC106N-VIS(/M) from Thorlabs. To account for the difference in pulse energy between the trilayer and the other materials (see Table SS1), the TA absorption and kinetic data are normalized to peak intensity.

	NIR (nJ)	VIS (nJ)
SWCNT	100	100
MoS_2/SWCNT	100	100
WSe_2/SWCNT	20	15
MoS_2/SWCNT/WSe_2	15	30

Table S1: Pulse energies associated with the TA spectral data for each material.

1.2 Additional Transient Absorption Spectra

Figure S1 shows the TA spectra of a WSe_2 bilayer using WSe_2 purchased from 2D semiconductors and WSe_2 grown via chemical vapor deposition at NREL. Upon selective excitation of WSe_2, electron or hole transfer do not occur, indicated by the absence of the WSe_2 ground state bleach (750 nm) and SWCNT trion induced absorption (1175 nm).

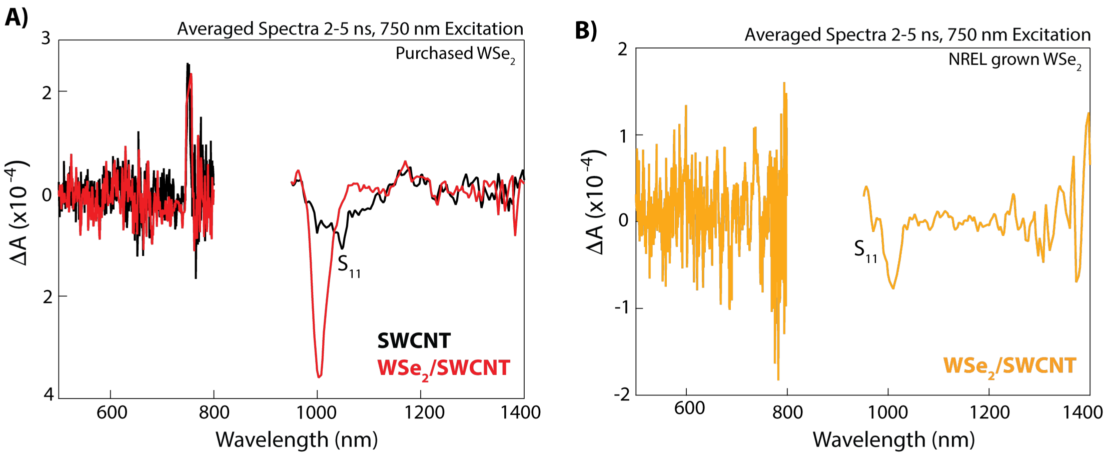

Figure S1: Transient absorption spectra averaged over 2-5 ns following 750nm excitation of A)SWCNT (black) and WSe_2/SWCNT bilayer (red) using purchased WSe_2 and; B) WSe_2/SWCNT bilayer using NREL grown WSe_2.

Figure S2 shows the transient spectra of neat WSe_2 after 750 nm excitation at 200 fs, 100 ps and 5 ns delay. The A, B and C exciton GSB can be osberved at 750 nm, 600 nm and 510 nm, respectively.

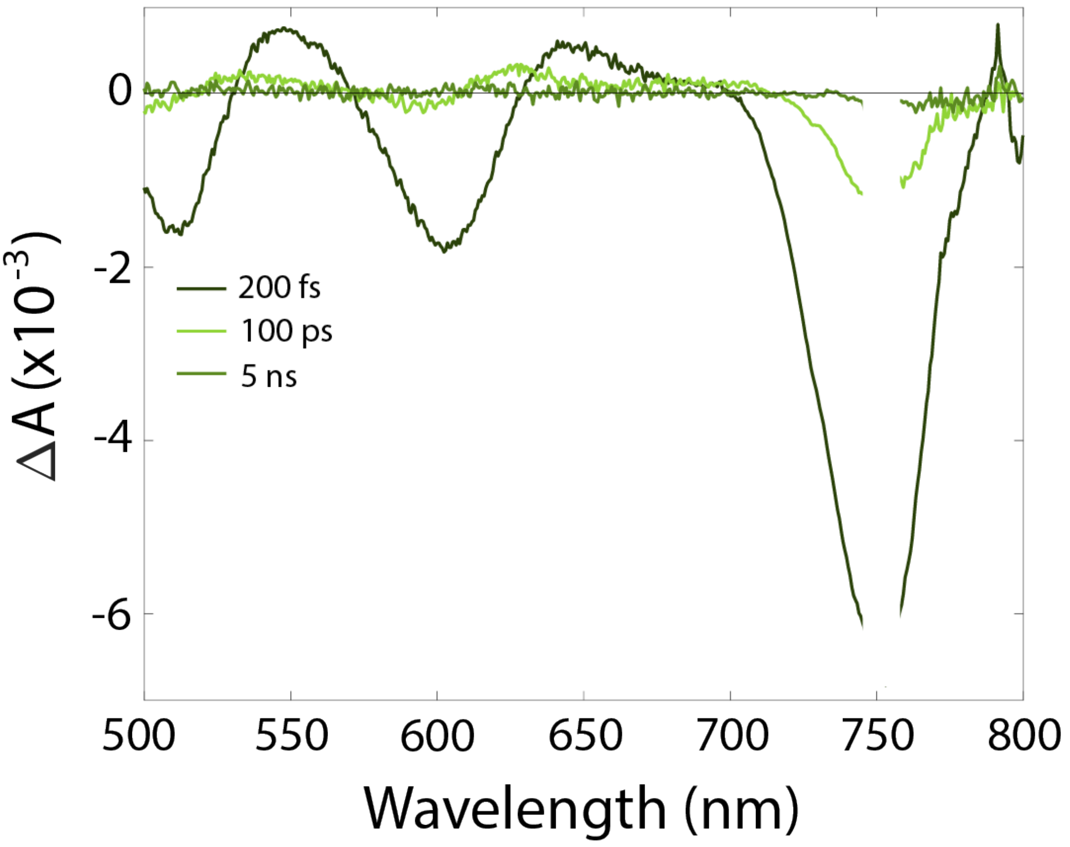

Figure S2: Transient absorption spectra of neat WSe_2 after 750 nm excitation. The A exciton GSB can be observed around 750 nm, B exciton at 600 nm and C exciton at 510 nm.

In the main text we speculate that releasing WSe_2 from its original substrate may modify the valence band energy in such a way that hole transfer is now spontaneous in the trilayer, but not in the bilayer. Figure S3 shows no presence of hole transfer in the "reverse" SWCNT/WSe_2 bilayer (SWCNTs spray coated onto monolayer WSe_2 grown via CVD directly on a sapphire substrate), refuting this hypothesis.

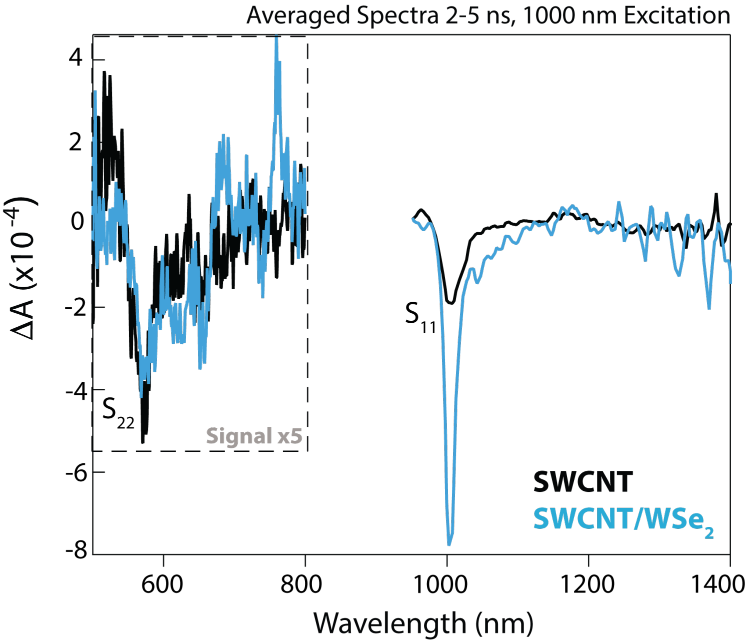

Figure S3: transient absorption spectra averaged over 2-5 ns following 1000nm excitation of SWCNT (black) and SWCNT/WSe_2 reverse bilayer.

Figure S4a shows the transient absorption spectra of the MoS_2/SWCNT bilayer and trilayer upon selective excitation of WSe_2 at 750 nm. When tracking the MoS_2 GSB, selective excitation of WSe_2 in the trilayer results in a slow rise of MoS_2 charges (Figure S4b).

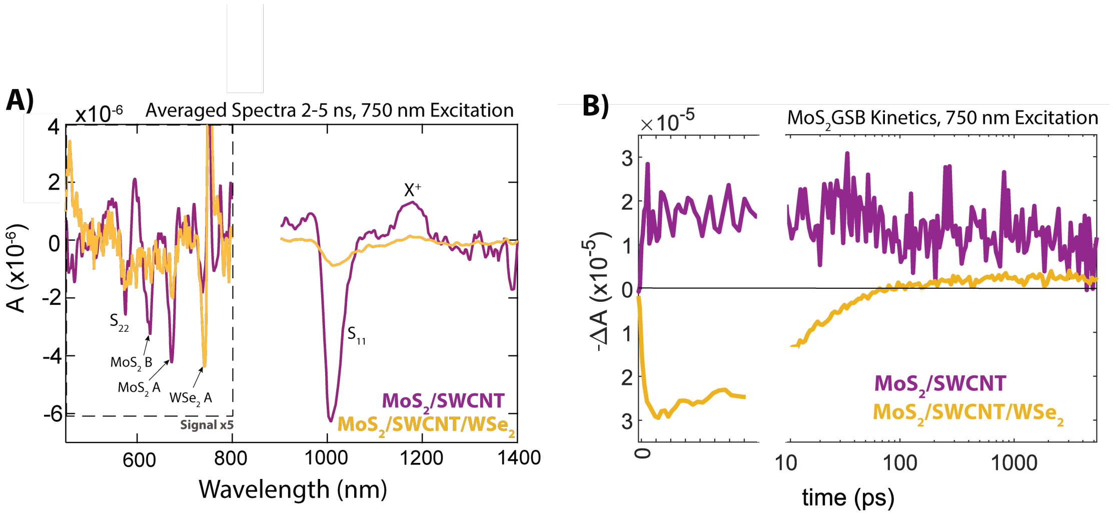

Figure S4: A)Transient absorption spectra averaged over 2–5 ns, MoS2/SWCNT bilayer (purple) and MoS2/SWCNT/WSe_2 trilayer (gold); B) Kinetic traces at 660 nm, corresponding to the ground state bleach of MoS_2 following 750 nm excitation.

Figure S5A shows the kinetic traces corresponding to the trion wavelength (1175 nm) for the MoS_2/SWCNT bilayer, MoS_2/SWCNT/WSe_2 trilayer and the simulation obtained from the curve fitting procedure. The initial decay comes from the initially populated singlet SWCNT state that gives rise to the charge separated states. The 200 fs delay spectra for the corresponding bilayer, trilayer and neat SWCNT are also present in panel B showing the initial contribution at 1175 nm that corresponds to singlet SWCNTs photoinduced absorption (PIA). Due to the use of the air free holder to prevent sample damage/degradation, there is reflection due to the air free holder walls. Figure S6 shows the presence of the second pulse in all our samples marked by the rectangle, and it shows the effect it has on the trion kinetics. Important to notice panel c where we show the "rise" of the trion signal due to presence of the second pulse, the "rise" is also present in the MoS_2 A exciton kinetics but not the artifact around 10 ps. From our fitting model for the trilayer (black curve in Figure S5A) you can observe that we do not model a rise of the trion feature because this is an artifact and not real kinetics present on the system of interest.

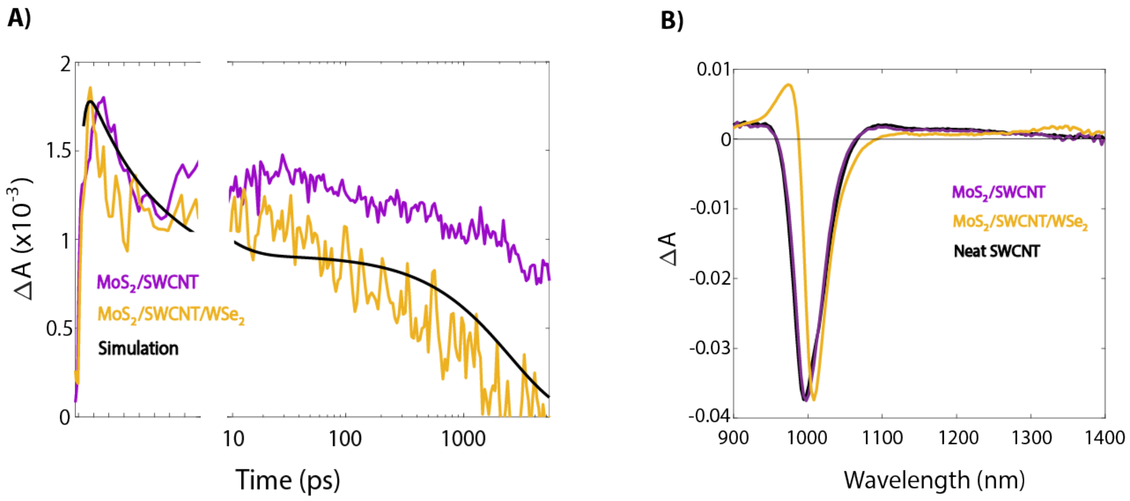

Figure S5: A)Trion kinetics for the MoS_2/SWCNT bilayer (purple), MoS_2/SWCNT/WSe_2 trilayer (gold) and the simulation obtained from our curve fitting procedure (black), highlighting that early time dynamics correspond to singlet contributions from direct SWCNT excitation and longer time dynamics are governed by the trion feature. B) Early time (200 fs) spectra for the neat SWCNT, MoS_2/SWCNT bilayer and MoS_2/SWCNT/WSe_2 trilayer showing the photoinduced absorption band is present due to singlet state contribution from direct SWCNT excitation.

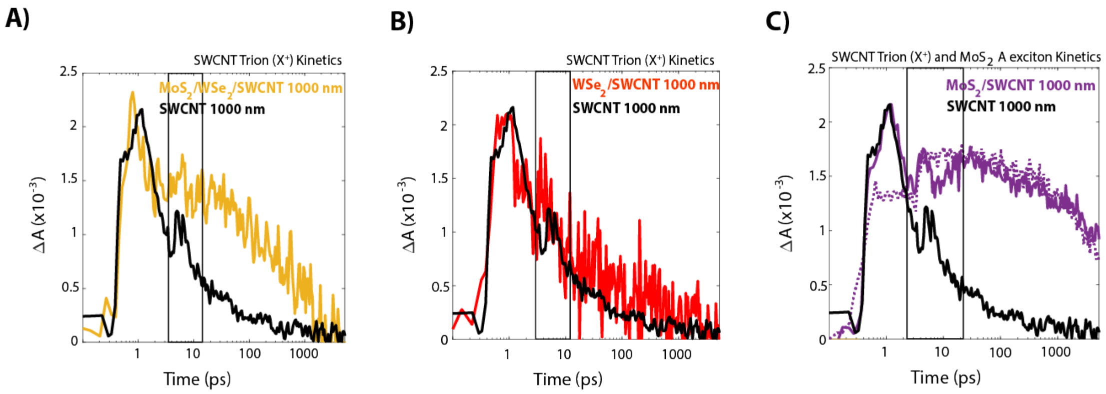

Figure S6: A) Trion kinetics for the MoS_2/SWCNT/WSe_2 trilayer (yellow) and neat SWCNT (black) showing the second pulse inside the marked area. A) Trion kinetics for the WSe_2/SWCNT bilayer (red) and neat SWCNT (black) showing the second pulse inside the marked area. C) Trion kinetics for the MoS_2/SWCNT bilayer (solid purple) and neat SWCNT (black) and Mo_2 A exciton kinetcs showing the second pulse inside the marked area.

Figure S7 highlights a faster decay of trions in the nanosecond time frame for the trilayer, relative to the bilayer, continuing to make a case for charges diffusing away from SWCNTs.

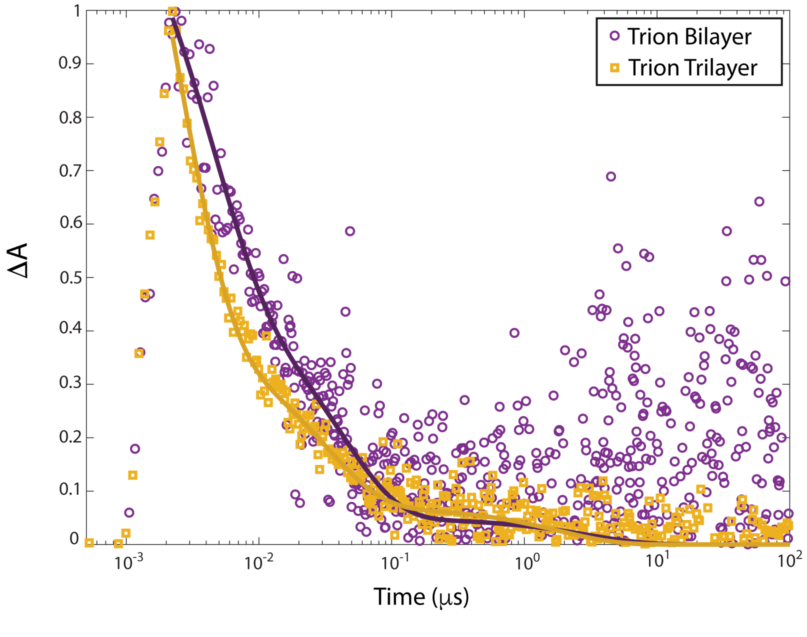

Figure S7: Amplitude averaged charge recombination lifetime in the MoS_2/SWCNT bilayer (purple) and MoS_2/SWCNT/WSe_2 trilayer (yellow).

Figure S8 compares the WSe_2/SWCNT bilayer transient spectroscopy results after 1000 nm and 750 nm excitation. Panel a shows the kinetics of the trion feature at 1175 nm, where we see a long time rise of the trion feature after 750 nm excitation while we observe only a decay after 1000 nm excitation. This hints to a different mechanism between electron transfer (750 nm, direct WSe_2 excitation) vs

hole transfer (1000 nm, direct SWCNT excitation). For future work, the difference in mechanism should be better studied. While in this work we focus only in direct SWCNT excitation to avoid multiple active pathways that make kinetic analysis more difficult.

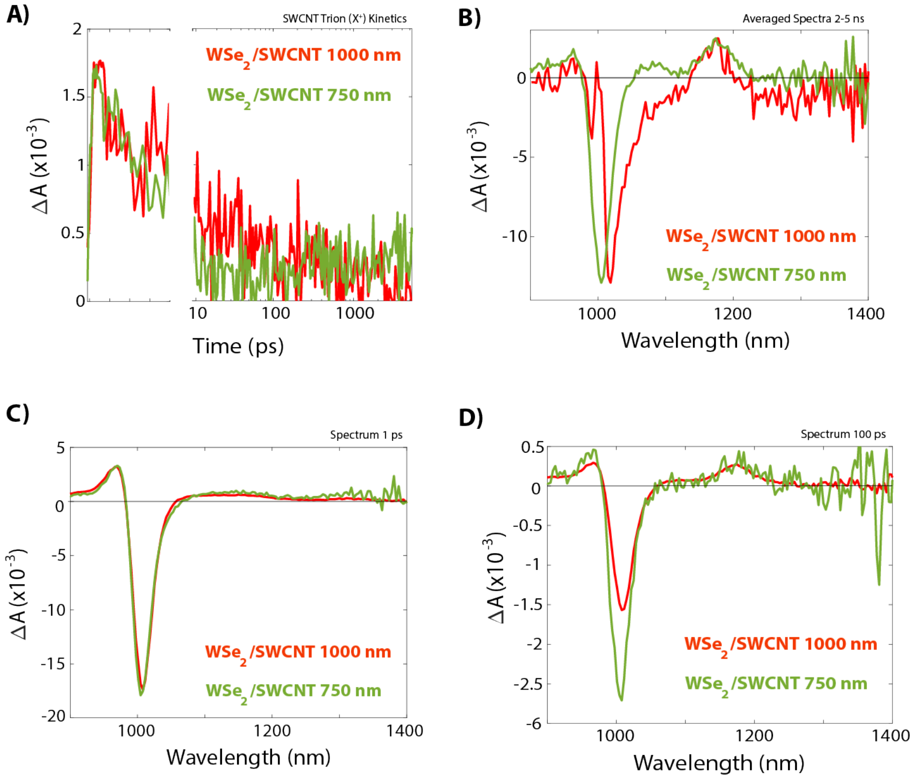

Figure S8: A) Kinetic traces corresponding to the SWCNT trion (X^+) induced absorption for the WSe_2/SWCNT bilayer after 1000 nm excitation (red) and 750 nm excitation (green). Transient absorption spectra for the WSe_2/SWCNT bilayer after 1000 nm excitation (red) and 750 nm excitation (green) B) averaged over 2–5 ns delay C) at 1 ps delay and D) at 100 ps delay.

1.3 Kinetic analysis

Multivariate curve resolution using Alternating Least Squares. A MATLAB package developed by Jaumot, et al, was used to perform initial singular value decomposition (SVD) and extract associated concentration profiles.[1,2] Visible and NIR regions were analyzed separately. The visible region was analyzed using four independent components based on initial TA information. The components correspond to the initial SWCNT excited state (S65*), electron transfer from SWCNT to MoS_2 ($S65^+$ MoS_2^-), hole transfer from SWCNT to WSe_2 ($S65^-$ WSe_2^+), and the charge separated state where SWCNT charges diffuse away from the initial SWCNT/TMDC generation site while the oppositely charged carriers remain in the respective TMDCs (MoS_2^- or WSe_2^+). Spectra for each component were fed to the MATLAB package as an initial guess from experimental TA. The kinetic scheme presented in Figure 5b is then proposed and the following rate equations specified:

$$\frac{dS_{65}^*}{dt} = -k_1 S_{65}^* \tag{1}$$

$$\frac{dMoS_2^- S_{65}^+ CS}{dt} = k_{11} S_{65}^* - k_2 MoS_2^- S_{65}^+ CS \tag{2}$$

$$\frac{dS_{65}^- WSe_2^+ CS}{dt} = k_{12} S_{65}^* - k_3 S_{65}^- WSe_2^+ CS \tag{3}$$

$$\frac{dMoS_2^- WSe_2^+ CS}{dt} = k_{31} S_{65}^- WSe_2^+ CS + k_{21} MoS_2^- S_{65}^+ CS - k_{rec} MoS_2^- WSe_2^+ CS \tag{4}$$

Solving these rate equations led to the following concentration equations, where the different rates are also shown in Figure 5a in the main text for reference:

$$S_{65}^* = S_{65}^*(0) e^{-k_1 t} \tag{5}$$

$$MoS_2^- S_{65}^+ CS = MoS_2^- S_{65}^+ CS(0) e^{-k_2 t} + \frac{k_{11} S_{65}^*(0)}{k_2 - k_1} [e^{-k_1 t} - e^{-k_2 t}] \tag{6}$$

$$S_{65}^- WSe_2^+ CS = S_{65}^- WSe_2^+(0) e^{-k_3 t} + \frac{k_{12} S_{65}^*(0)}{k_2 - k_1} [e^{-k_1 t} - e^{-k_3 t}] \tag{7}$$

$$\begin{aligned} MoS_2^- WSe_2^+ CS = {} & \frac{MoS_2^- S_{65}^+ CS(0) k_{21}}{k_2} (1 - e^{-k2t}) \\ & + \frac{k_{11} S_{65}^*(0)}{k_2 - k_1} [\frac{1 - e^{-k_1 t}}{k_1} - \frac{1 - e^{-k_2} t}{k_2}] + \frac{S_{65}^- WSe_2^+ CS(0) k_{31}}{k_3} (1 - e^{-k3t}) + \\ & \frac{k_{12} S_{65}^*(0)}{k_3 - k_1} [\frac{1 - e^{-k_1 t}}{k_1} - \frac{1 - e^{-k_3} t}{k_3}] \end{aligned} \tag{8}$$

Concentration profiles obtained from the MATLAB package are then fitted to the solved equations (5-8) until convergence is achieved, also done through the MATLAB package. Upon convergence, one of the outputs of the software is the associated spectrum to each component, shown below in Figure S1. The SWCNT initial excited state ($S65^*$, black) only presents the GSB for the S_{22} excitonic transition around 575 nm, the electron transfer from SWCNT to MoS_2 ($S65^+$ MoS_2^-, blue) presents the A and B exciton GSB for MoS_2 around 660 and 620 nm respectively, and the GSB of the SWCNT. The hole transfer from SWCNT to WSe_2 ($S65^-$ WSe_2^+, red) presents the A exciton GSB of WSe_2 around 740 nm and the SWCNT GSB, the B exciton GSB of WSe_2 is observed as a slight dip in the photo induced absorption around 600 nm. Lastly, the charge separated state generated by SWCNT charges diffusing away from the initial TMDC/SWCNT generation into the opposite TMDC layer (MoS_2^- WSe_2^+, green) presents all previously mentioned GSB features.

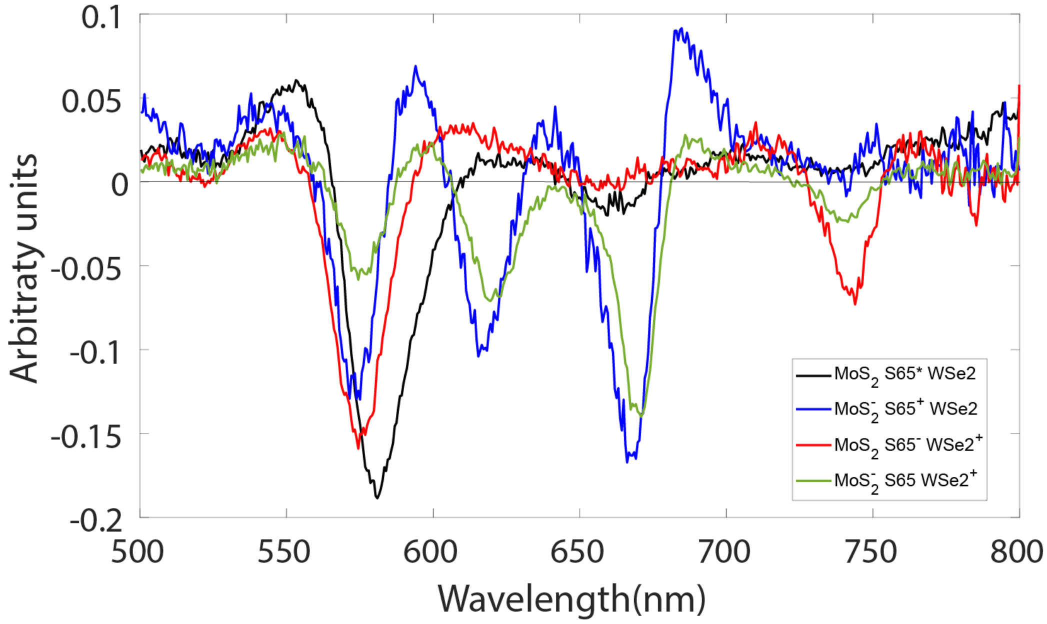

Figure S9: Associated spectra for the four different components obtained from the solved equations fitted to the concentration profiles obtained from the MATLAB package. SWCNT initial excited state (S65*, black), electron transfer from SWCNT to MoS_2 ($S65^+$ MoS_2^-, blue), hole transfer from SWCNT to WSe_2 ($S65^-$ WSe_2^+, red), and charge separated state generated by SWCNT charges diffusing away from the initial TMDC/SWCNT generation into the opposite TMDC layer (MoS_2^- WSe_2^+, green).

The concentration profiles are also an output from the MATLAB software shown in Figure 5c in the main text. From the fitted concentration profiles important kinetic information is obtained such as the rates for the different pathways presented in Table SS2 with the corresponding standard deviation. The decay for the last state (charges diffusing within the SWCNT layer) is not probed in ultrafast TA, as the limit of the stage is 5 ns. From a separate nanosecond-microsecond TA measurement, the longest decay of MoS_2 A exciton GSB was fitted to be around 7 µs (amplitude-averaged lifetime of 1.3 µs). A surface plot of the experimental TA data presented in Figure S10a is compared to the simulated TA data (Figure S10b) obtained from the fitted concentration equations and spectra are shown. The kinetic scheme and derived equations explain 95% of the experimental data.

The NIR region was analyzed with the same MATLAB package, based on the rate equations and solved concentration profiles for the visible region. Instead of four independent components, three components were specified because the electron transfer from SWCNT to MoS_2 ($S65^+$ MoS_2^-), and hole transfer from SWCNT to WSe_2 ($S65^-$ WSe_2^+) do not have different spectral signature in the NIR. These two components were included as one, resulting in a combined concentration profile. The solved concentration profiles, initially obtained from the visible region analysis were fed to the software resulting in the final output presented in Figure S11a, and the associated spectra shown in Figure S11b. The SWCNT initial excited state (S65*, black) only presents the GSB for the S_{11} excitonic transition around 1010 nm. The electron transfer from SWCNT to MoS_2 ($S65^+$ MoS_2^-) combined with the hole transfer from SWCNT to WSe_2 ($S65^-$ WSe_2^+) presents the trion (X^+) feature

around 1180 nm and the SWCNT GSB (purple). Lastly, the charge separated state formed by charge diffusion within the SWCNT layer to the opposite TMDC (MoS_2^- WSe_2^+, green) presents remaining SWCNT GSB because the charges have left the SWCNTs to inject (via diffusion-limited CT) into the opposite TMDC.

From the concentration profiles and associated spectra, the quantum yields for electron and hole transfer were calculated. The associated spectrum for electron transfer from SWCNT to MoS_2 ($S65^+$ MoS_2^-), and hole transfer from SWCNT to WSe_2 ($S65^-$ WSe_2^+) cannot be indistinguishable in the NIR but from the visible region independent concentration profiles have been obtained, allowing independent calculation of carrier density for each state. The hole transfer quantum yield is 0.29, and electron transfer quantum yield is 0.12, accounting for a total charge transfer yield of 0.41. This derivation and calculation is further explained in section 3 below. A surface plot of the experimental TA data presented in Figure S12a is compared to the simulated TA data (Figure S12b) obtained from the fitted concentration equations and spectra are shown. The kinetic scheme and derived equations explain 95% of the experimental data.

	s^{-1}	Std. dev
k_1	$6.5{*}10^{11}$	$1.4{*}10^{11}$
k_2	$6.7{*}10^{8}$	$2.0{*}10^{8}$
k_3	$4.5{*}10^{11}$	$5.0{*}10^{10}$
k_{11}	$6.5{*}10^{11}$	$1.4{*}10^{11}$
k_{12}	$> 6.5 * 10^{11}$	—
k_{21}	$4.0{*}10^{8}$	$2.0{*}10^{8}$
k_{rec}^1	$2.7{*}10^{8}$	$2.8{*}10^{8}$
k_{31}	$2.0{*}10^{11}$	$2.0{*}10^{10}$
k_{rec}^2	$2.5{*}10^{11}$	$2.8{*}10^{10}$
k_{rec}^3	$8.1{*}10^{5}$	$4.9{*}10^{4}$

Table S2: Rate constants associated with the kinetic scheme

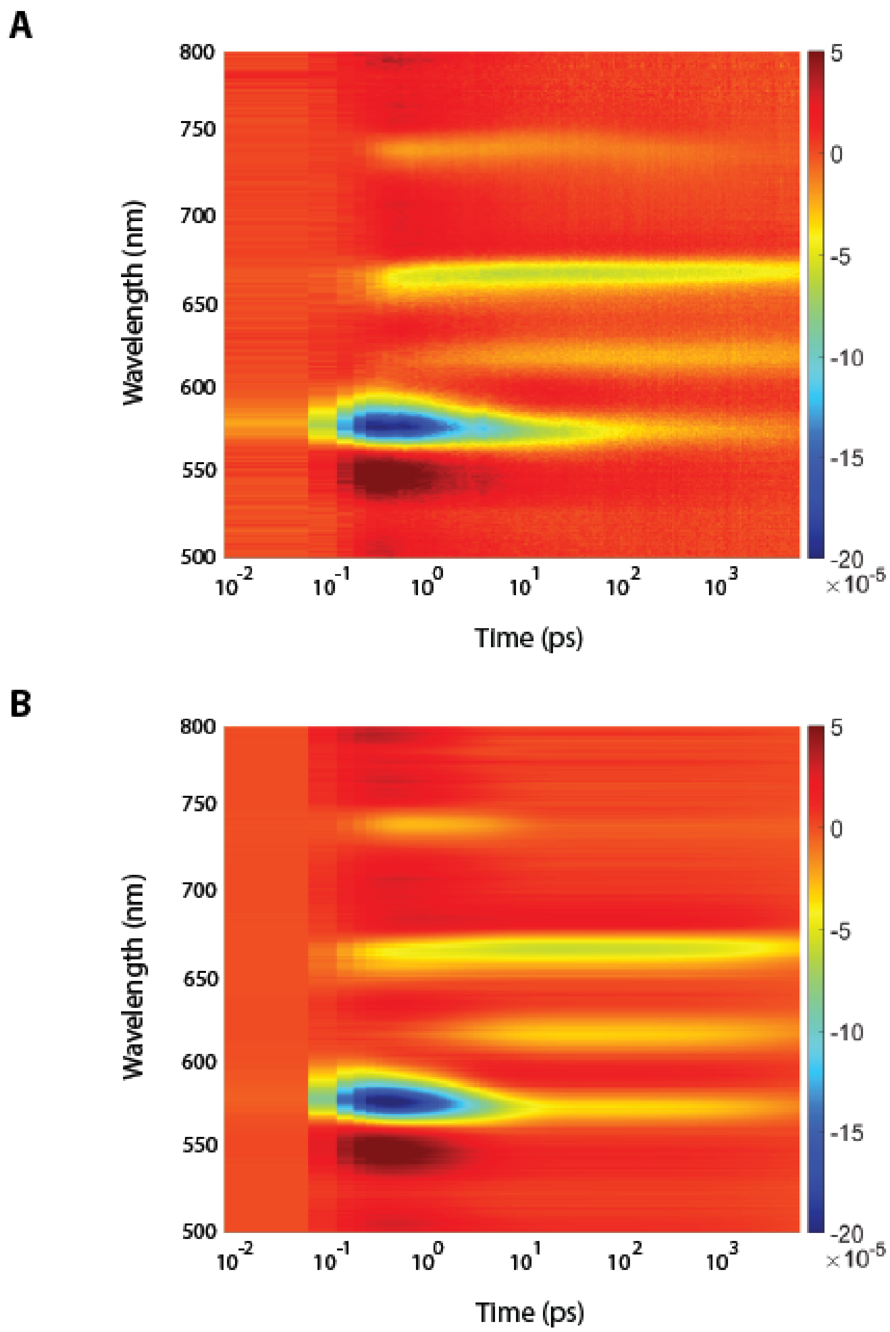

Figure S10: A) Experimental TA surface plot for the visible region of the trilayer excited at 1000 nm. B) Simulated TA surface plot from the concentration equations and associated spectra. Vertical axis is wavelength (nm): GSB for S_{22} at 575 nm, MoS_2 A exciton at 660 nm and WSe_2 at 740 nm can be identified. Horizontal axis is time (ps), showing the behavior along time. Color-bar to the right specifies intensities of the different signals

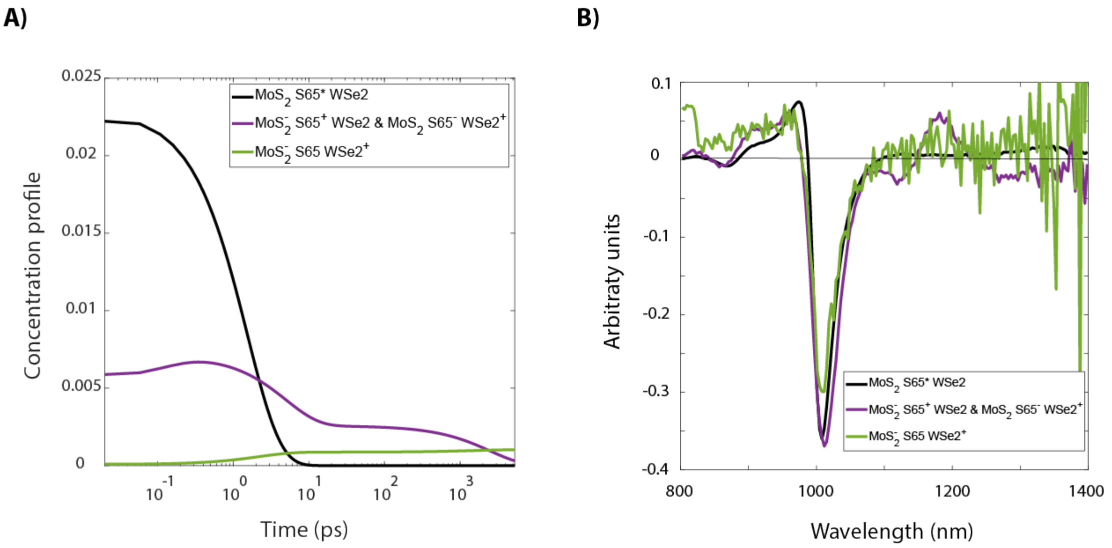

Figure S11: A) Concentration profile for the three NIR components obtained from MATLAB package after convergence. B) Associated spectra for the three different NIR components obtained from the solved equations fitted to the concentration profiles obtained from the MATLAB package. SWCNT initial excited state (S65*, black), electron transfer from SWCNT to MoS_2 ($S65^+$ MoS_2^-) combined with hole transfer from SWCNT to WSe_2 ($S65^-$ WSe_2^+) (purple curve), and charge separated formed by diffusion-limited charge transfer after the initial ultrafast charge transfers (MoS_2^- WSe_2^+, green).

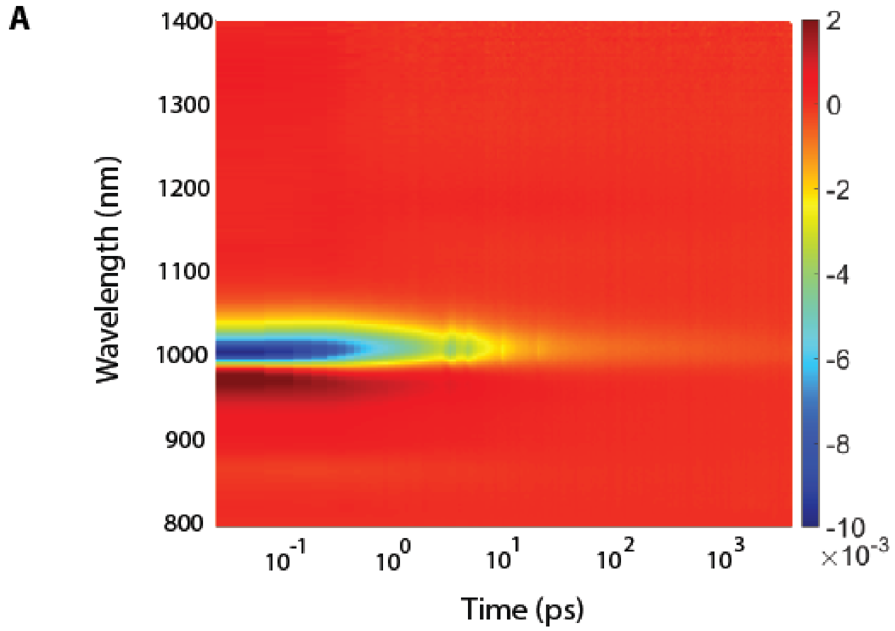

Figure S12: A) Experimental TA surface plot for the NIR region of the trilayer excited at 1000 nm. B) Simulated TA surface plot from the concentration equations and associated spectra. Vertical axis is wavelength (nm): GSB for S_{11} at 1010 nm can be identified. Horizontal axis is time (ps), showing the behavior along time. Colorbar to the right specifies intensities of the different signals

2 Calculating ΔG

The Gibbs free energy change for electron and hole transfer between WSe_2 - SWCNT and SWCNT - MoS_2 was calculated using the equation:

$$\Delta G_{ET/HT} = |IP_D - EA_A| - E_{opt,D/opt,A} \tag{9}$$

The electron affinity (EA) and ionization potential (IP) for (6,5) SWCNTs are taken as -3.86 eV and -5.40 eV respectively.[3] The EA and IA for MoS_2 in Table S3 are an average of two calculated band energies and one experimental value for MoS_2 on sapphire.[4] The optical band gap of WSe_2 and MoS_2 are calculated from the A exciton in their respective absorption spectra (see Figure 1). The electronic band gap (E_{el}) can be calculated from the sum of the optical band gap (E_{opt}) and the binding energy of the exciton ($E_{b,exc}$).[4] Using the calculated optical band gap and exciton binding energy the electronic band gap for WSe_2 and MoS_2 are 1.89 ev and 1.9 eV respectively.

$$E_{el} = E_{opt} + E_{b,exc} \tag{10}$$

	EA (eV)	IP (eV)	E_{opt} (eV)	$E_{b,exc}$ (eV)	E_{el} (eV)
MoS_2	-4.23	-6.13	1.87	0.24	2.11
WSe_2	-3.2	-5.02	1.65	0.24	1.89
SWCNT	-3.86	-5.4	1.24	0.3	1.54

Table S3: A summary of electron affinity (EA), ionization potential (IP), optical band gap (E_{opt}), binding energy ($E_{b,exc}$) and electronic band gap (E_{el}) for MoS_2, WSe_2 and SWCNT used to calculate ΔG.[3–5]

3 Charge Transfer Yield

The S_{11} optical density ($OD_{S_{11}}$) is the maximum of the S_{11} absorption peak in the trilayer (see Figure 1e). The trion ($OD_{X+/-}$) optical density is from the TA spectra associated with the concentration profiles where both the WSe_2 and MoS_2 charge concentration are at their max. The relative trion optical density ($OD_{X+}/OD_{S_{11}}$) is used to find the hole or electron concentration according to Dowgiallo et al.:

$$\frac{OD(X^+)}{OD(S_{11})} = 0.0674 - 0.0676e^{-21.5N_{h/e}} \tag{11}$$

where $N_{h/e}$ is the hole or electron density/nm of tube length. We then determine the charge yield as $N_{h/e}/N_{exc}$, where N_{exc} is the initial exciton concentration from the number of absorbed photons in the pump area. The parameters used to calculate N_{exc} are found in Table S4 and S5.[6–8]

beam area	$2.022x10^{-3}cm^2$
energy fluence	$1.5x10^{-8}J$
incident energy fluence	$7.41x10^{-6}$ J/cm^2
excitation wavelength	1000 nm
energy of singlet photon	$1.98x10^{-19}$ J/photon
incident Photon fluence	$3.74x10^{13}$ $photons/cm^2$
S_{11} trilayer absorbance	0.029
absorbed photon fluence	$2.43x10^{12}$ $photons/cm^2$
areal density of C atoms	$6.76x10^{15}$ C $atoms/cm^2$
nm $tube/cm^2$	$7.46x10^{13}$ nm $tube/cm^2$
excitons/nm tube	0.032 exciton/nm tube
$OD(X^+)_{MoS_2}$	$3.43x10^{-4}$
$OD(X^-)_{WSe_2}$	$1.56x10^{-4}$
OD (S11)	0.029
charge yield	41%

Table S4: Summary of values used to calculate excitons/nm of tube, relative trion optical density and charge transfer yield for the MoS_2/SWCNT/WSe_2 trilayer excited at 1000 nm at 15 nJ fluence

Parameter	1000 nm	750 nm
beam area	$2.022x10^{-3}cm^2$	$5.348x10^{-3}cm^2$
energy fluence	$2.0x10^{-8}J$	$1.5x10^{-8}$
incident energy fluence	$9.89x10^{-6}$ J/cm^2	$2.80x10^{-6}$ J/cm^2
excitation wavelength	1000 nm	750 nm
energy of singlet photon	$1.98x10^{-19}$ J/photon	$2.65x10^{-19}$ J/photon
incident photon fluence	$4.99x10^{13}$ $photons/cm^2$	$1.06x10^{13}$ $photons/cm^2$
trilayer absorbance at corresponding wavelength	0.092	0.015 (WSe_2)<br>0.009 (SWCNT)
absorbed photon fluence	$9.57x10^{12}$ $photons/cm^2$	$3.60x10^{11}$ $photons/cm^2$
areal density of C atoms	$6.76x10^{15}$ C $atoms/cm^2$	$6.76x10^{15}$ C $atoms/cm^2$
nm $tube/cm^2$	$7.46x10^{13}$ nm $tube/cm^2$	$7.46x10^{13}$ nm $tube/cm^2$
excitons/nm tube	0.128 exciton/nm tube	$4.82x10^{-3}$ exciton/nm
$OD(X^{-/+})_{WSe_2}$	$3.35x10^{-4}$	$6.18x10^{-5}$
OD (S11)	0.092	0.092
charge yield	HT = 2.0%	ET = 10%

Table S5: Summary of values used to calculate excitons/nm of tube, relative trion optical density and charge transfer yield for the WSe_2/SWCNT bilayer excited at 1000 nm at 20 nJ fluence and 750 nm at 15 nJ fluence

References

(1) Jaomut, J.; Gargallo, R.; de Juan, A.; Tauler, R. A graphical user-friendly interface for MCR-ALS: a new tool for multivariate curve resolution in MATLAB. *Chemometr. Intell. Lab. Syst.* **2005**, *76*, 101–110.

(2) Jaomut, J.; de Juan, A.; Tauler, R. MCR-ALS GUI 2.0: new features and applications. *Chemometr. Intell. Lab. Syst.* **2015**, *140*, 1–12.

(3) Kang, H. S.; Sisto, T. J.; Peurifoy, S.; Arias, D. H.; Zhang, B.; Nuckolls, C.; Blackburn, J. L. Long-lived charge separation at heterojunctions between semiconducting single-walled carbon nanotubes and perylene diimide electron acceptors. *J. Phys. Chem. C.* **2018**, *122*, 14150–14161.

(4) Sulas-Kern, D. B.; Miller, E. M.; Blackburn, J. L. Photoinduced charge transfer in transition metal dichalcogenide heterojunctions – towards next generation energy technologies. *Energy Environ. Sci* **2020**, *13*, 2684–2740.

(5) Blackburn, J. L. Semiconducting single-walled carbon nanotubes in solar energy harvesting. *ACS Energy Lett.* **2017**, *2*, 1598–1613.

(6) Sulas-Kern, D. B.; Zhang, H.; Li, Z.; Blackburn, J. L. Microsecond charge separation at heterojunctions between transition metal dichalcogenide monolayers and single-walled carbon nanotubes. *Mater. Horiz.* **2019**, *6*, 2103–2111.

(7) Dowgiallo, A.-M.; Mistry, K. S.; Johnson, J. J.; Reid, O. G. Probing exciton diffusion and dissociation in single-walled carbon nanotube-C_{60} heterojunctions. *J.Phys. Chem. Lett.* **2016**, *7*, 1794–1799.

(8) Ferguson, A. J.; Reid, O. G.; Nanayakkara, S. U.; Ihly, R.; Blackburn, J. L. Efficiency of charge-transfer doping.in organic semiconductors probed with quantitative microwave and direct-current conductance. *J. Phys. Chem. Lett.* **2018**, *9*, 6864–6870.
